# Recurrent Pelvic Lipoblastoma With Sciatic Nerve Compression in an Infant: A Case Report

**DOI:** 10.7759/cureus.110139

**Published:** 2026-06-02

**Authors:** Soufia Charaf, Kenza Elmkaddem, Sanaa Bouramdane, Sarra Benmiloud

**Affiliations:** 1 Department of Pediatric Hematology-Oncology, Hassan II University Hospital, Fez, MAR; 2 Faculty of Medicine, Pharmacy, and Dentistry, Sidi Mohamed Ben Abdellah University, Fez, MAR

**Keywords:** infant, lipoblastoma, pelvic lipoblastoma, sciatic nerve compression, soft tissue tumor

## Abstract

Lipoblastoma is an uncommon benign tumor derived from embryonal adipose tissue and is most commonly diagnosed in children under three years of age. Pelvic localization is uncommon and may lead to compressive complications depending on tumor size and extension. We report the case of a 17-month-old male infant presenting with a left pelvic lipoblastoma with gluteal extension. The clinical history was marked by progressive limping evolving over six months. Clinical examination revealed a pelvic mass associated with neurological impairment of the left lower limb. MRI demonstrated a large fatty tumor with extra-pelvic extension and compression of the sciatic nerve. Histopathological examination confirmed the diagnosis of lipoblastoma. Complete surgical excision was performed. The postoperative course was marked by local recurrence five months later, requiring a second surgical excision, with no further recurrence during follow-up. Pelvic lipoblastoma is a rare entity that may present with neurological complications related to mass effect. MRI plays a central role in preoperative evaluation and postoperative surveillance, whereas histopathological examination confirms the diagnosis. Complete surgical excision is the mainstay of treatment.

## Introduction

Lipoblastoma is a rare benign tumor arising from embryonal adipose tissue, occurring predominantly in infants and children younger than three years of age [1]. It differs from other lipomatous tumors by the presence of immature adipocytes at various stages of differentiation [2]. Lipoblastoma most commonly occurs in the extremities and trunk, whereas deep localizations, particularly pelvic involvement, remain uncommon [1,3]. Depending on its size and extension, the tumor may cause compressive manifestations related to mass effect, including neurological complications [2,3]. The diagnosis mainly relies on imaging, especially MRI, although deep pelvic lipoblastoma may radiologically mimic other adipocytic tumors, including malignant entities such as myxoid liposarcoma, making preoperative diagnosis challenging [2]. Definitive confirmation is based on histopathological examination. Surgical excision remains the treatment of choice; however, the risk of recurrence justifies prolonged follow-up [1,2]. Pelvic lipoblastoma associated with sciatic nerve compression and neurological impairment remains exceptionally rare in the literature, highlighting the diagnostic and therapeutic challenges related to deep pelvic extension.

We report the case of a pelvic lipoblastoma in an infant complicated by sciatic nerve involvement and early local recurrence.

## Case presentation

We report the case of a 17-month-old male infant with no significant past medical history, presenting with a left pelvic mass extending to the ipsilateral gluteal region. The patient presented with progressive limping for six months without any history of trauma. An initial consultation with a traumatologist, performed two months after symptom onset, suggested a post-traumatic etiology. One month later, due to persistent symptoms, a pediatric evaluation revealed a left pelvic mass. Spinal and pelvic MRI was performed before referral to our institution.

On admission, physical examination revealed a left-sided pelvic mass extending to the ipsilateral gluteal region, measuring approximately 6 × 6 cm, without overlying inflammatory signs. The umbilical circumference was 43 cm, and the abdominal circumference was 46 cm. Neurological examination demonstrated motor weakness of the left lower limb associated with ipsilateral muscle wasting and decreased deep tendon reflexes, particularly the Achilles reflex.

Spinal and pelvic MRI demonstrated a large, well-circumscribed left pelvic fatty mass with lobulated contours centered within the obturator compartment. The lesion showed hyperintense signal on both T1- and T2-weighted sequences with signal attenuation on fat-suppressed images and contained internal septa with low T2 signal intensity. The mass measured 83 × 50 mm in its largest axial dimensions and extended extra-pelvically into the subcutaneous soft tissues of the left gluteal region. The lesion caused compression of the pelvic portion of the sciatic nerve. Although lipoma and myxoid liposarcoma were considered in the radiological differential diagnosis, the patient’s age and the presence of a well-circumscribed, lobulated fatty mass with high signal intensity on both T1- and T2-weighted images favored lipoblastoma (Figure 1).

**Figure 1:**
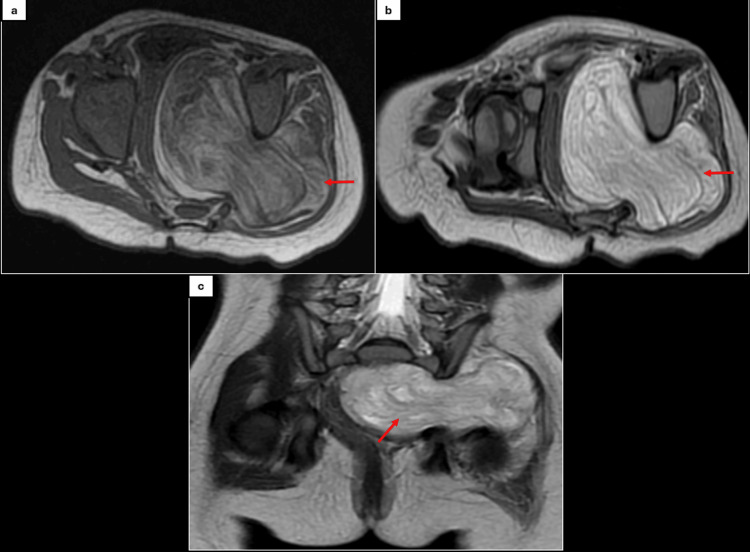
Pelvic MRI demonstrating a large left pelvic fatty mass centered within the obturator compartment Axial T1-weighted (a) and T2-weighted (b) images show a well-circumscribed hyperintense lesion containing internal hypointense septa (arrow). Coronal MRI image (c) demonstrates extra-pelvic extension into the subcutaneous soft tissues of the left gluteal region through an enlarged ipsilateral obturator foramen. The lesion measured approximately 83 × 50 mm in its largest axial dimensions (arrow).

Tumor biopsy revealed a well-circumscribed multilobulated proliferation composed of adipocytic cells at different stages of maturation, ranging from primitive mesenchymal cells to mature adipocytes, with intervening lipoblasts within a focally myxoid stroma, compatible with lipoblastoma. Immunohistochemical and molecular analyses, including PLAG1 immunohistochemistry and fluorescence in situ hybridization, were not available at our institution; therefore, the diagnosis was established based on the histopathological findings in conjunction with the clinical and radiological presentation. Electroneuromyography confirmed involvement of the left sciatic nerve.

Complete surgical excision was performed. Resection was macroscopically and microscopically complete (R0). Histopathological examination of the surgical specimen confirmed the diagnosis of lipoblastoma. Molecular analysis for PLAG1 rearrangement was not performed. The postoperative course was initially marked by progressive neurological improvement with recovery of motor function following rehabilitation therapy.

Five months after surgery, local tumor recurrence was identified on follow-up pelvic MRI, which demonstrated a recurrent left pelvic mass measuring approximately 47 × 57 × 32 mm, with extra-pelvic extension into the subcutaneous soft tissues of the gluteal region, in close contact with the sciatic nerve but without evidence of nerve compression (Figure 2). No neurological deficit was observed at the time of recurrence.

**Figure 2:**
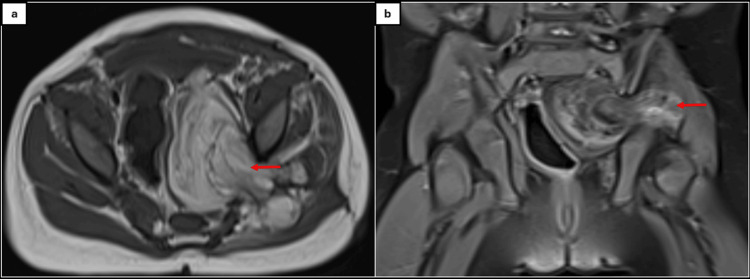
Follow-up pelvic MRI demonstrating local recurrence of the lipoblastoma Axial image (a) shows a recurrent left pelvic fatty mass measuring approximately 47 × 57 × 32 mm. Coronal image (b) demonstrates extra-pelvic extension into the subcutaneous soft tissues of the left gluteal region (arrows).

A second surgical excision of the fatty mass was successfully performed without complications. The subsequent outcome was favorable, with no postoperative complications, neurological impairment, or evidence of recurrence after two months of follow-up.

## Discussion

Lipoblastoma is a rare benign adipocytic soft tissue tumor composed of immature fat cells resulting from abnormal embryonal development of white adipose tissue [2]. It accounts for less than 1% of all childhood tumors and shows a male predominance, with a reported male-to-female ratio of approximately 1.5:1 [3]. Lipoblastoma most frequently involves the extremities but may also occur in less common sites such as the head and neck, trunk, mediastinum, kidney, mesentery, retroperitoneum, and perineum [3]. Pelvic localizations complicated by neurological involvement remain exceptionally uncommon, with only limited cases reported in the literature [4]. The tumor mainly affects infants and young children and may cause compressive complications related to mass effect, particularly when located in deep or anatomically complex regions [5].

Morphologically, two forms were described: a localized, encapsulated, well-circumscribed form corresponding to lipoblastoma and a diffuse, multicentric, nonencapsulated form known as lipoblastomatosis, characterized by infiltration of adjacent tissues [3]. Clinically, lipoblastoma usually presents as a painless and progressively enlarging mass that may remain unnoticed for several months [6]. However, in deep localizations, symptoms depend on the mass effect exerted on adjacent structures and may lead to functional manifestations, including neurological deficits in compressive forms, as illustrated in our patient [2].

Radiologically, ultrasonography is generally the first imaging modality performed and typically demonstrates a well-defined mass that may appear heterogeneous with internal septations or cystic components. However, its diagnostic performance remains limited because of variable imaging appearances [7]. MRI is considered the imaging modality of choice, allowing accurate evaluation of tumor extension and anatomical relationships [7]. Advanced MRI sequences such as diffusion-weighted imaging may provide additional information for the characterization of adipocytic tumors and their differential diagnosis [2]. In our patient, MRI was the initial imaging examination performed, and the radiological findings were consistent with those reported in the literature, where lipoblastoma typically appears as a well-circumscribed lobulated mass with heterogeneous hyperintense signal on both T1- and T2-weighted sequences. Nevertheless, imaging findings alone may not reliably distinguish lipoblastoma from other adipocytic tumors, particularly lipoma and myxoid liposarcoma [8].

Definitive diagnosis relies on histopathological examination, which demonstrates proliferation of adipocytic cells at different stages of maturation associated with a myxoid stroma and a characteristic capillary network [6,7]. Histopathological findings in our case were similar and supported the diagnosis. Additional immunohistochemical or cytogenetic analyses, including PLAG1 rearrangement testing, may be useful to differentiate lipoblastoma from malignant tumors such as liposarcoma [2,9]. However, molecular testing for PLAG1 rearrangement was not available at our institution. In our patient, management was based on complete surgical excision, which remains the standard treatment. Complete resection with negative margins is recommended to reduce the risk of recurrence. However, deep tumor localization may complicate the surgical approach and limit the possibility of complete excision [10].

Despite its benign nature, lipoblastoma carries a non-negligible risk of local recurrence estimated at approximately 15%, particularly in cases of incomplete excision or diffuse forms [4,10]. This risk justifies prolonged postoperative surveillance [4,5]. The early recurrence observed in our patient highlights the importance of close clinical and radiological follow-up, mainly based on MRI, to detect recurrence early and assess tumor extension. A follow-up duration of at least five years, and up to 10 years in some cases, has been recommended because of the possibility of late recurrence [10]. In our case, the postoperative follow-up after the second surgery remains relatively short, and continued long-term surveillance is therefore required. However, despite its recurrence potential, lipoblastoma remains a benign tumor with no reported malignant transformation or metastatic potential [11].

## Conclusions

Lipoblastoma is a rare pediatric tumor that may present atypically in cases of deep pelvic localization with neurological compressive manifestations. Diagnosis relies on a combination of imaging findings and histopathological confirmation. Surgical management aims for complete excision; however, the risk of recurrence requires prolonged clinical and radiological surveillance. This case highlights the importance of considering lipoblastoma in the differential diagnosis of deep fatty pelvic masses in children and emphasizes the need for careful long-term follow-up to improve outcomes. Early recognition of neurological symptoms and multidisciplinary long-term follow-up are essential, particularly in recurrent cases with deep pelvic extension.
